# WS_2_ and MoS_2_ biosensing platforms using peptides as probe biomolecules

**DOI:** 10.1038/s41598-017-10221-4

**Published:** 2017-08-31

**Authors:** Xiuxia Sun, Jun Fan, Caihong Fu, Linyan Yao, Sha Zhao, Jie Wang, Jianxi Xiao

**Affiliations:** 1 State Key Laboratory of Applied Organic Chemistry, Key Laboratory of Nonferrous Metal Chemistry and Resources Utilization of Gansu Province, College of Chemistry and Chemical Engineering, Lanzhou University, Lanzhou, 730000 P. R. China; 2 Key laboratory of Magnetic Resonance in Biological Systems, State Key Laboratory of Magnetic Resonance and Atomic and Molecular Physics, Wuhan Institute of Physics and Mathematics, Chinese Academy of Sciences, Wuhan, 430071 China

## Abstract

Biosensors based on the two-dimensional layered nanomaterials transition metal dichalcogenides such as WS_2_ and MoS_2_ have shown broad applications, while they largely rely on the utilization of single stranded DNA as probe biomolecules. Herein we have constructed novel WS_2_- and MoS_2_- based biosensing platforms using peptides as probe biomolecules. We have revealed for the first time that the WS_2_ and MoS_2_ nanosheets display a distinct adsorption for Arg amino acid and particularly, Arg-rich peptdies. We have demonstrated that the WS_2_ and MoS_2_ dramatically quench the fluorescence of our constructed Arg-rich probe peptide, while the hybridization of the probe peptide with its target collagen sequence leads to the fluorescence recovery. The WS_2_-based platform provides a sensitive fluorescence-enhanced assay that is highly specific to the target collagen peptide with little interferences from other proteins. This assay can be applied for quantitative detection of collagen biomarkers in complex biological fluids. The successful development of WS_2_- and MoS_2_- based biosensors using non-ssDNA probes opens great opportunities for the construction of novel multifunctional biosensing platforms, which may have great potential in a wide range of biomedical field.

## Introduction

Graphene-based nanomaterials have shown broad biosensing applications due to their fascinating properties such as water dispersibility, biocompatibility and versatile surface modification^1–6^. The selective adsorption of graphene oxide (GO) with single stranded DNA (ssDNA) has led to the design of a variety of fluorescent biosensors^7–11^. The hybridization of dye-labeled ssDNA with complementary DNA sequences has been used to develop efficient assays for a number of DNA targets^7–11^. The strong noncovalent binding between the dye-labeled ssDNA and target proteins has also been proved as a valuable approach to detect various proteins^12^. Furthermore, the adsorption of aromatic and positively charged amino acids onto GO has inspired scientists to construct peptide probes using GO as a highly specific sensing platform^6, 13, 14^.

Transition metal dichalcogenides such as WS_2_ and MoS_2_ are newly discovered two-dimensional layered nanomaterials analogous to graphene^15, 16^. They have aroused extensive attention due to their unique optical and catalytic properties^15, 17^. Compared with graphene, transition metal dichalcogenides nanosheets can be facilely synthesized without the need of oxidation treatment, which is a critical process for graphene oxide preparation^17–19^. The oxidation degree has a huge impact on the fluoresence quenching efficiency of GO, indicating that the performance of transition metal dichalcogenides nanosheets are more controllable. Furthermore, it has been reported that thiolated compounds can be added to the surfaces of WS_2_ nanosheets by simple self-assembly process^20^. These discoveries imply that transition metal dichalcogenides nanosheets may have very promising biomedical applications.

MoS_2_ nanosheets have recently been discovered to be able to adsorb ssDNA by the van der Waals force between nucleobases and the basal plane of MoS_2_ nanosheets^19–24^. The adsorption of dye-labeled ssDNA onto MoS_2_ leads to fluorescence quenching^24–26^, while the hybridization between ssDNA and its complementary target DNA sequence results in much weaker interaction with MoS_2_ and therefore recovers the fluorescence^21, 23, 26, 27^. The fluorescence intensity of the system provides a quantitative indication of the target DNA sequence^21, 23^. By using assorted aptamers, this strategy has been utilized to detect small molecules, microRNAs and proteins^27^. However, only ssDNA has been used as probe molecules in these MoS_2_-based detection platforms till date. The interaction between transition metal dichalcogenides nanosheets and other biomolecules such as amino acids, peptides and proteins remains little understood. The coupling of transition metal dichalcogenides nanosheets with other biomolecules may provide a powerful tool to develop novel biosensors with versatile features and promising applications in a wide range of biomedical field.

Collagen, the most abundant structural protein, provides a molecular scaffold for the human body^28–31^. The degradation of collagen triple helix by matrix metalloproteinases is involved in many biological processes such as tissue development and regeneration as well as a variety of pathological conditions including arthritis and tumors^32–34^. Therefore, it is of utmost importance to develop efficient assays to detect the degraded products of collagen. Current techniques mainly rely on the successful development of antibodies^35–40^. However, the highly repetitive (Gly-X-Y)_n_ amino acid sequence of collagen makes it very challenging to produce antibodies with high specificity. Furthermore, these approaches often suffer from serious drawbacks such as tedious and time-consuming procedures, and low binding affinities^35–40^. It remains an urgent need to construct simple methods to detect collagen biomarkers.

**Figure 1:**
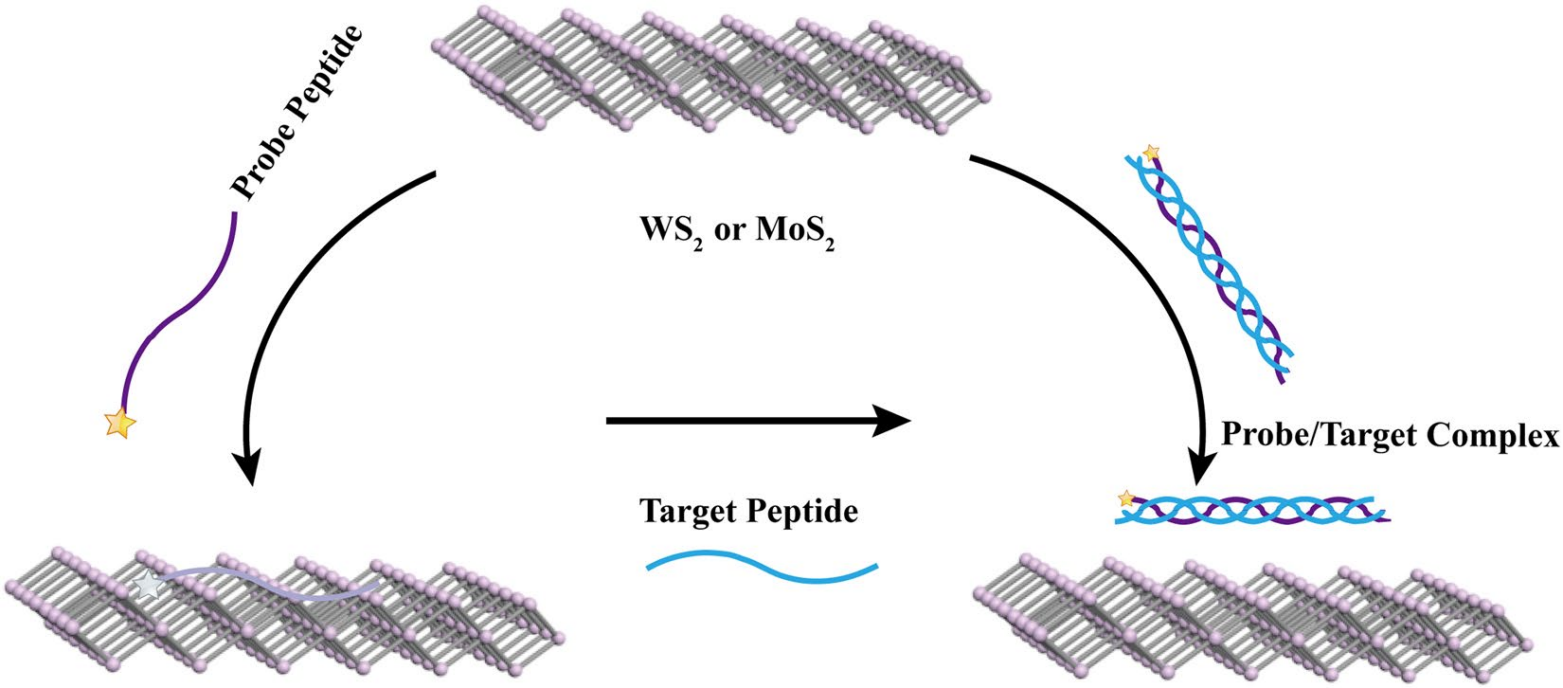
Schematic Illustration of novel WS_2_- and MoS_2_- based biosensing platforms using peptides as probe biomolecules to specifically recognize the target. The strong adsorption of the fluorescent probe peptide by WS_2_ or MoS_2_ quenches the fluorescence, while the hybridization of the probe and target molecules results in fluorescence recovery.

Herein we have designed novel WS_2_- and MoS_2_- based biosensing platforms using peptides as probe biomolecules, and demonstrated their applications for the detection of collagen. The WS_2_ and MoS_2_ nanosheets show a strong adsorption of the constructed fluorescent probe peptide, leading to the quenching of the fluorescence of the dye. The hybridization of the probe and target peptides removes the probe peptide from the WS_2_ and MoS_2_, thus recovering the fluorescence (Fig. 1). We have discovered for the first time that the WS_2_ and MoS_2_ nanosheets display a significant adsorption for Arg amino acid and Arg-rich peptdies. We have further demonstrated that the coupling of the WS_2_ and MoS_2_ platforms with our constructed Arg-rich probe peptide provides a highly specific and sensitive assay for target collagen biomarkers in complex biological fluids.

## Materials and Methods

### Materials

Commercial tungsten disulfide (WS_2_) and molybdenum disulfide (MoS_2_) nanomaterials was obtained from Gezhi New Materials Co., Ltd. (Anqing, China). BSA (Albumin bovine V), myoglobin, hemoglobin and protamine sulfate were obtained from Sangon Biotech (Shanghai, China). All the reagents obtained from commercial sources were of analytical grade and were used without further purification. Ultrapure water was used to prepare all the solutions. All the experiments were repeated for three times.

### Instrumentation

The FT-IR spectra of WS_2_ and MoS_2_ were obtained on a Nicolet Nexus 670 infrared spectrophotometer (Madison, Wisconsin). Raman spectra were measured at room temperature on a Renishaw InVia Raman Microscope with an excitation laser at 633 nm (NCNST). TEM micrographs were characterized using a FEI TECNAIG^2^ transmission electron microscope (Hillsborough, Oregon). Fluorescence measurements were carried out using a RF-5301PC fluorescence spectrometer (Shimadzu Instruments Ltd., Japan).

### Peptide synthesis

Peptides POG (with amino acid sequence G(POG)_10_) and FAM-PRG (with amino acid sequence 5-FAM-G(PRGPOG)_5_; FAM, 5-Carboxyfluorescein) were synthesized by Chinese Peptide Company (Hangzhou, China). Other peptides FAM-5G (with amino acid sequence FAM-GGGGG), FAM-5R (with amino acid sequence FAM-RRRRR), FAM-POG (with amino acid sequence FAM-G(POG)_10_) were synthesized in-house by standard Fmoc solid phase synthesis method^41^. Briefly, the peptides were synthesized on 2-chlorotrityl chloride resin at a 0.1 mmol scale. Stepwise couplings of amino acids were performed by a double coupling method using Fmoc-amino acids (4 eq.), DIEA (6 eq.), HBTU (4 eq.) and HOBt (4 eq.). After each step of coupling, the reaction mixture was washed with DMF (3 × 3.5 mL) and DCM (3 × 3.5 mL), and 20% piperidine in DMF was then added to remove the Fmoc protection group. Test reagent (2% ethanal DMF, 2% chloranil DMF) was applied to monitor the completion of coupling reaction and Fmoc deprotetction every cycle. FAM was added at the N-terminal of the peptide by using FAM (12 eq.), DIEA (6 eq.), HBTU (4 eq.) and HOBt (4 eq.) under gentle shaking for 18 hrs at room temperature. TFA/H2O (95:5) was used to cleave the peptides from the resin and remove the tBu groups. The peptides were harvested by precipitation with cold Et_2_O, and crude products were collected after re-suspension in cold Et_2_O, sonication and centrifugation. These peptides were purified using reverse phase HPLC on a C18 column, and the purity of the peptides were confirmed by mass spectrometry.

### Investigation of the interaction of amino acids with WS_2_ and MoS_2_ nanosheets

The interaction of 19 different amino acids with WS_2_ and MoS_2_ nanosheets were investigated. A mixture solution of amino acids (Asp, Thr, Ser, Glu, Cys, Gly, Ala, Cys-Cys, Met, Ile, Leu, Tyr, Phe, His, Trp, Lys, Arg, Hyp, Pro) was incubated with 1 mg/mL WS_2_ nanosheets in PBS buffer at 25 °C for 10 hrs under gentle shaking. The mixture was then centrifuged at 13,000 rpm for 30 min to collect the supernatants. The concentrations of the tested amino acids before and after incubation with the WS_2_ nanosheets were analyzed using Hitachi L-8900 high speed amino acid analyzer following the standard protocol. The concentration ratio of each amino acid after and before incubation with the WS_2_ nanosheets was then calculated. The concentration ratio of 19 amino acids after and before incubation with the MoS_2_ nanosheets was measured similarly. Each measurement was performed three times.

### Investigation of the interaction of fluorescent labeled peptides with WS_2_ and MoS_2_ nanosheets

The interaction of fluorescent labeled peptides with WS_2_ and MoS_2_ nanosheets were investigated, respectively. The solutions of FAM, FAM-5G, FAM-5R, FAM-POG, and FAM-PRG at the same concentration of 1 μM were prepared, respectively; and their fluorescence intensities were measured on a RF-5301PC fluorescence spectrometer (Shimadzu Instruments Ltd, Japan). 53.3 μg/mL WS_2_ was then added to each solution, and their fluorescence intensities were recorded. The same measurements were also performed for FAM, FAM-5G, FAM-5R, FAM-POG, and FAM-PRG using MoS_2_ nanosheets (53.3 μg/mL).

### WS_2_- and MoS_2_- based assays using fluorescent labeled peptides for the detection of target collagen peptide

The fluorescent labeled peptide FAM-PRG was further investigated to develop WS_2_- and MoS_2_- based assays for the detection of target collagen peptide POG. Peptide FAM-PRG was dissolved in 20 mM PBS (pH 7.4), and incubated at 90 °C for 20 min to make it in an unfolded single stranded state prior to any experiments. The fluorescence spectra were recorded after different concentrations of WS_2_ (0–86.7 μg/mL) was added to the solution of the probe peptide FAM-PRG (1 µM) with an excitation wavelength of 497 nm. Standard solutions of the target collagen peptide POG were prepared by serial dilution.

In the fluorescence assays of the target collagen peptide POG, a “pre-mixing” method was employed due to the slow dynamics of the triple helix formation for collagen mimic peptides^30^. The probe peptide FAM-PRG (300 µM) was incubated with the target peptide POG (600 µM) in 50 µL PBS buffer at 90 °C for 20 min and then at 4 °C for >24 hrs to ensure the successful hybridization between the probe and target peptides (at a molar ratio of 1:2). The hybridized mixture was then diluted 300 times to obtain a final concentration of peptide FAM-PRG as 1 µM. The WS_2_ and MoS_2_ solution with a final concentration of 53.3 µg/mL was added into the mixture for fluorescence measurements. PBS buffers at different pHs (5.6, 6.0, 7.4, 8.0 and 9.0) were prepared to evaluate the pH effect on fluorescence recovery efficiency.

For the quantitative assay, different final concentrations of the target peptide POG (25, 50, 100, 200, 300, and 400 nM) were hybridized with the probe peptide FAM-PRG, and the fluorescence spectra were recorded after the addition of WS_2_. The urine and saliva samples were provided by laboratory personnel. The supernatants of samples were harvested after centrifugation at 1000 rpm for 10 min, and diluted to 20% by 20 mM PBS buffer (pH 7.4). The hybridized FAM-PRG/POG mixtures with different final concentration of peptide POG (25, 50, 100, 200, and 300 nM) were added in the biological media, and the fluorescence spectra were measured after the addition of WS_2_. Protein BSA, myoglobin, hemoglobin and protamine sulfate with a final concentration of 2 µM were added to investigate the interference of other proteins in the detection of the target collagen peptide POG. All the measurements were repeated for 3 times.

## Results and Discussion

### Characterization of WS_2_ and MoS_2_

The obtained WS_2_ and MoS_2_ nanomaterials were characterized by FT-IR, Raman and TEM techniques (Fig. S1). The FT-IR spectrum of WS_2_ showed intense peaks at 3426 cm^−1^ (O-H stretching) and 1622 cm^−1^ (water bonding) (Fig. S1A), while the FT-IR spectrum of MoS_2_ showed intense peaks at 3457 cm^−1^ (O–H stretching) and 1615 cm^−1^ (water bonding) (Fig. S1D)^42^. These FT-IR spectra represented the characteristic vibrations of WS_2_ and MoS_2_ and matched well with previous reports^42^. The Raman spectrum of WS_2_ showed a strong peak assigned to the vibration mode of E^1^_2g_ at 352 cm^−1^ and another well-documented strong peak assigned to the vibration of A_1g_ at 418 cm^−1^ (Fig. S1B)^43, 44^. The Raman spectrum of MoS_2_ showed similar peaks at 372 cm^−1^ (E^1^_2g_ mode) and 401 cm^−1^ (A_1_g mode) (Fig. S1D), indicating the high purity of the synthesized nanomaterials^20, 45, 46^. The TEM micrographs showed typical layered structures of WS_2_ and MoS_2_, respectively (Fig. S1C and F)^20, 45, 46^. The characteristic FT-IR, Raman and TEM graphs confirmed the obtained nanomaterials as WS_2_ and MoS_2_, respectively.

### Investigation of the interaction of amino acids with WS_2_ and MoS_2_ nanosheets

**Figure 2:**
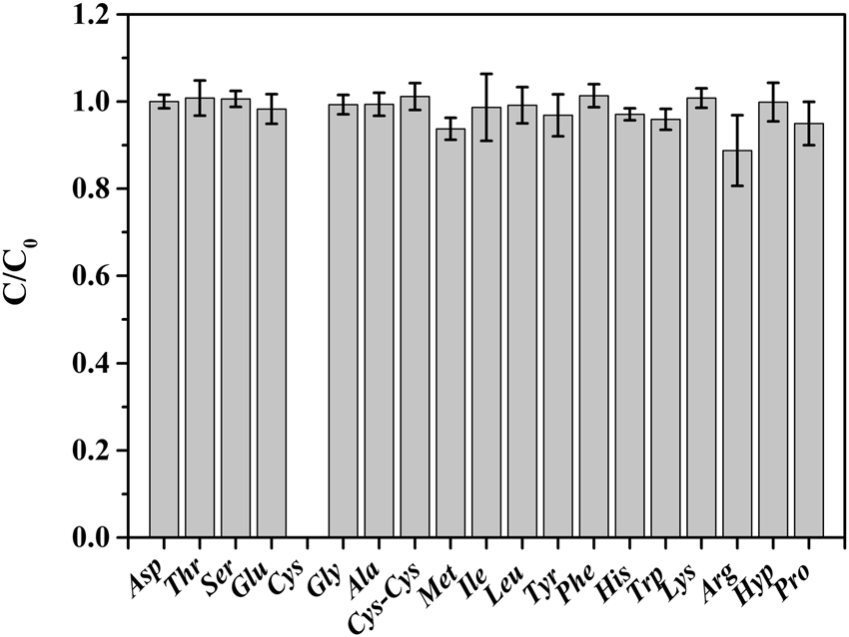
The concentration ratio (C/C_o_) of the tested amino acids after and before the incubation with WS_2_. C is the concentration of the tested amino acids after the incubation with WS_2_, representing the un-absorbed amino acid by WS_2_; C_o_ is the concentration of the tested amino acids before the incubation with WS_2_, representing the total amino acid.

In order to construct novel WS_2_- and MoS_2_- based sensing platforms using peptides as probe biomolecules, we first evaluate the interaction of amino acids with the WS_2_ and MoS_2_ nanosheets. The adsorption of 19 different amino acids with WS_2_ and MoS_2_ nanosheets were analyzed using Hitachi L-8900 high speed amino acid analyzer following the standard protocol. The concentration ratio of each amino acid after and before incubation with the WS_2_ nanosheets was then calculated (Fig. 2). Most amino acids showed a concentration ratio value of 1, demonstrating that they were likely not to be significantly adsorbed by the WS_2_. Most notably, the ratio for free Cys was zero, indicating that Cys could be 100% attached to the WS_2_ nanosheets; instead, the ratio of Cys-Cys was nearly 1, demonstrating that the Cys without free thiol group cannot be bound to the surface of the WS_2_ nanosheets (Fig. 2). This was consistent with earlier report that the surfaces of WS_2_ nanosheets can be easily modified by thiolated compounds through the simple self-assembly process^27^. Distinctively, besides Cys, another amino acid Arg showed the second smallest concentration ratio (0.89) for the WS_2_ nanosheet. Considering the relatively uniform ratio value of 1 for most amino acids, the smaller ratio value suggested that some Arg may be adsorbed to the WS_2_ (Fig. 2).

Similar situations were also observed for the adsorption of amino acids with the MoS_2_ nanosheets (Fig. S2). The concentration ratio for free Cys was zero, indicating a strong adsorption of Cys by the MoS_2_ nanosheets. Amino acid Arg also showed relatively smaller ratio values than most amino acids, suggesting that some Arg may be adsorbed to the MoS_2_ (Fig. S2). To conclude, we have for the first time discovered that Arg, in addition to the previously reported Cys, may show a distinct adsorption onto the WS_2_ and MoS_2_ nanosheets. With the aim to build WS_2_- and MoS_2_- based peptide biosensor, we therefore focus on the construction of Arg-containing peptides.

### Investigation of the interaction of fluorescent labeled peptides with WS_2_ and MoS_2_ nanosheets

**Figure 3:**
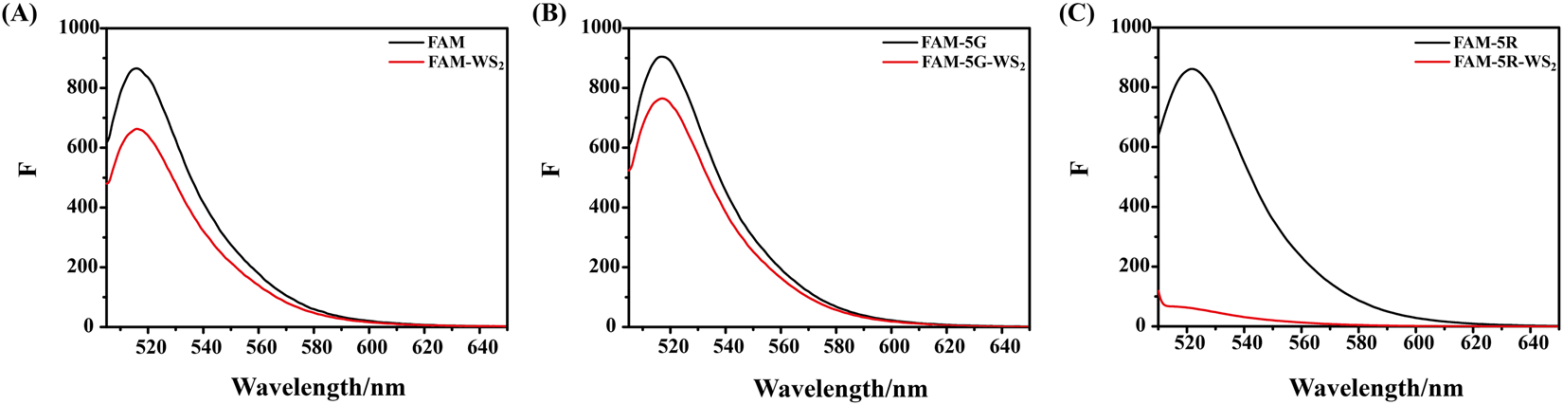
The fluorescence quenching of FAM, FAM-5G and FAM-5R by WS_2_. The fluorescence intensities of FAM (**A**), FAM-5G (**B**) and FAM-5R (**C**) were measured in the presence (red) and absence (black) of WS_2_ (**A**–**C**).

We further investigate if the inclusion of multiple Arg in peptides can enhance the interaction between peptides and WS_2_ nanosheets by evaluating the quenching efficiency of fluorescent labeled peptides by WS_2_ (Fig. 3). The FAM dye alone showed strong fluorescence, while the addition of the WS_2_ nanosheets quenched the fluorescence of the dye by a small degree (Fig. 3A). The fluorescence of peptide FAM-5G was also only quenched by the WS_2_ nanosheets by a small extent, indicating that the conjugation of 5 Gly with the FAM dye may not help the binding of the peptide to the WS_2_ nanosheets (Fig. 3B). In contrast, the fluorescence of peptide FAM-5R is hugely quenched by the WS_2_ nanosheets, indicating that the coupling of 5 Arg with the FAM dye greatly enhanced the binding of the peptide with the WS_2_ nanosheets (Fig. 3C). Similar cases were also observed for the MoS_2_ nanosheets (Fig. S3). The fluorescence of FAM and peptide FAM-5G was only slightly quenched by the MoS_2_ nanosheets, while the fluorescence of peptide FAM-5R was hugely quenched (Fig. S3). These results indicated that the inclusion of multiple Arg greatly enhanced the adsorption of the peptide onto the MoS_2_ as well as WS_2_ (Fig. S3).

**Figure 4:**
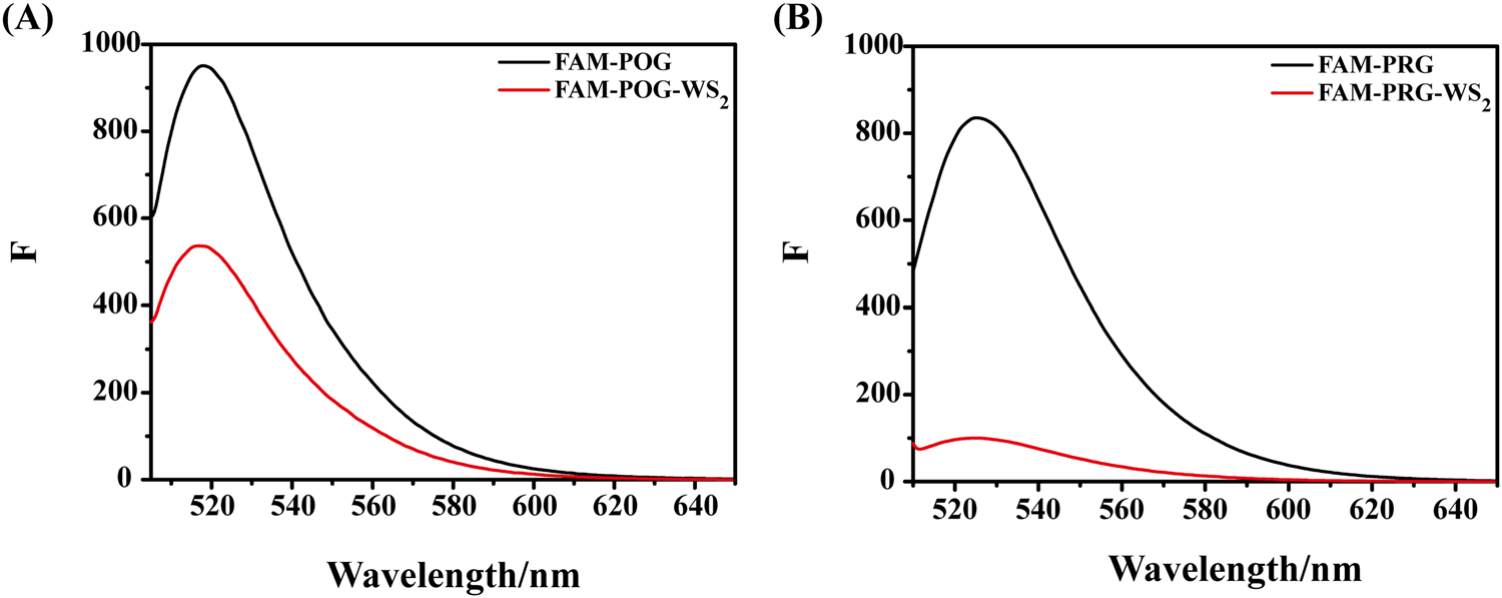
The fluorescence quenching of FAM-POG and FAM-PRG by WS_2_. The fluorescence intensities of FAM-POG (**A**), and FAM-PRG (**B**) were measured in the presence (red) and absence (black) of WS_2_ (**A**,**B**).

With the aim to develop assays for collagen detection, two collagen mimic peptides were also tested (Fig. 4). For the WS_2_ nanosheets, peptide FAM-POG showed strong fluorescence, which could be partially quenched by WS_2_ (Fig. 4A); while the strong fluorescence of peptide FAM-PRG could be almost totally quenched by WS_2_ (Fig. 4B). Similarly, the MoS_2_ nanosheets showed much stronger ability to quench the fluorescence of peptide FAM-PRG rather than FAM-POG (Fig. S4). Peptide FAM-PRG was constructed to contain 5 more Arg than peptide FAM-POG. All these results convincingly demonstrated that the inclusion of amino acid Arg promotes the interaction of peptide with WS_2_ and MoS_2_ nanosheets, leading to more dramatic fluorescence quenching.

### WS_2_- and MoS_2_- based fluorescence assays using the Arg-rich probe peptide FAM-PRG

Our future characterizations focused on the Arg-rich probe peptide FAM-PRG in order to develop an efficient assay for collagen biomarkers. We first optimized the conditions for WS_2_ to quench the fluorescence of the probe peptide. The fluorescence emission spectra were recorded for peptide FAM-PRG in the single stranded state after the addition of various concentrations of WS_2_ (0–86.7 µg/mL) (Fig. S5). The probe peptide FAM-PRG in the absence of WS_2_ showed strong fluorescence emission at 524 nm due to the presence of the fluorescein-based dye (Fig. S5). The fluorescence intensity of FAM-PRG gradually decreased when an increased amount of WS_2_ was added. When the concentration of WS_2_ reached 53.3 µg/mL, it resulted in ~93% quenching of the fluorescence of FAM-PRG. It suggested that WS_2_ behaved as an excellent quencher for the probe peptide.

We further investigated whether the WS_2_/FAM-PRG platform can be used to detect target collagen sequences. The fluorescence emission spectra were obtained for the probe peptide FAM-PRG hybridized or unhybridized with the target collagen sequence POG in the presence of WS_2_ at pH 7.4 in 20 mM PBS buffer (Fig. S6A). The fluorescence of peptide FAM-PRG was totally quenched by WS_2_, while the hybridization of FAM-PRG with the target peptide POG hugely increased the fluorescence (Fig. S6A). It suggested that the hybridization of peptide FAM-PRG with POG resulted in the formation of triple helix structure, which showed much weaker interaction with WS_2_. The MoS_2_ showed a similar situation that the hybridization of the probe and target peptides restored the fluorescence, indicating that both WS_2_ and MoS_2_ could be used as sensing platforms for collagen sequences (Fig. S6B).

Our development of the assay was then focused on the WS_2_ platform. The fluorescence restoration capability of peptide POG was further investigated under different pHs (Fig. S7A). The quenching efficiency of the probe peptide FAM-PRG by WS_2_ showed some changes under different pHs. The addition of POG on the WS_2_/FAM-PRG platform resulted in strong fluorescence restoration at pH 7.4, pH 8.0 and pH 9.0, while the fluorescence recovery was hugely reduced at acidic pH 5.6 and pH 6.0. In addition, the amount of extra salt (20 mM, 50 mM, 100 mM, 150 mM, 200 mM and 500 mM) affected the fluorescence quenching by some extent, while they showed little effect on the restoration level (Fig. S7B). The effect of pH and the salt on the quenching efficiency may suggest the presence of electrostatic interactions between the highly charged probe peptide and WS_2_.

### Detection of the target collagen sequence POG by the WS_2_/FAM-PRG platform

**Figure 5:**
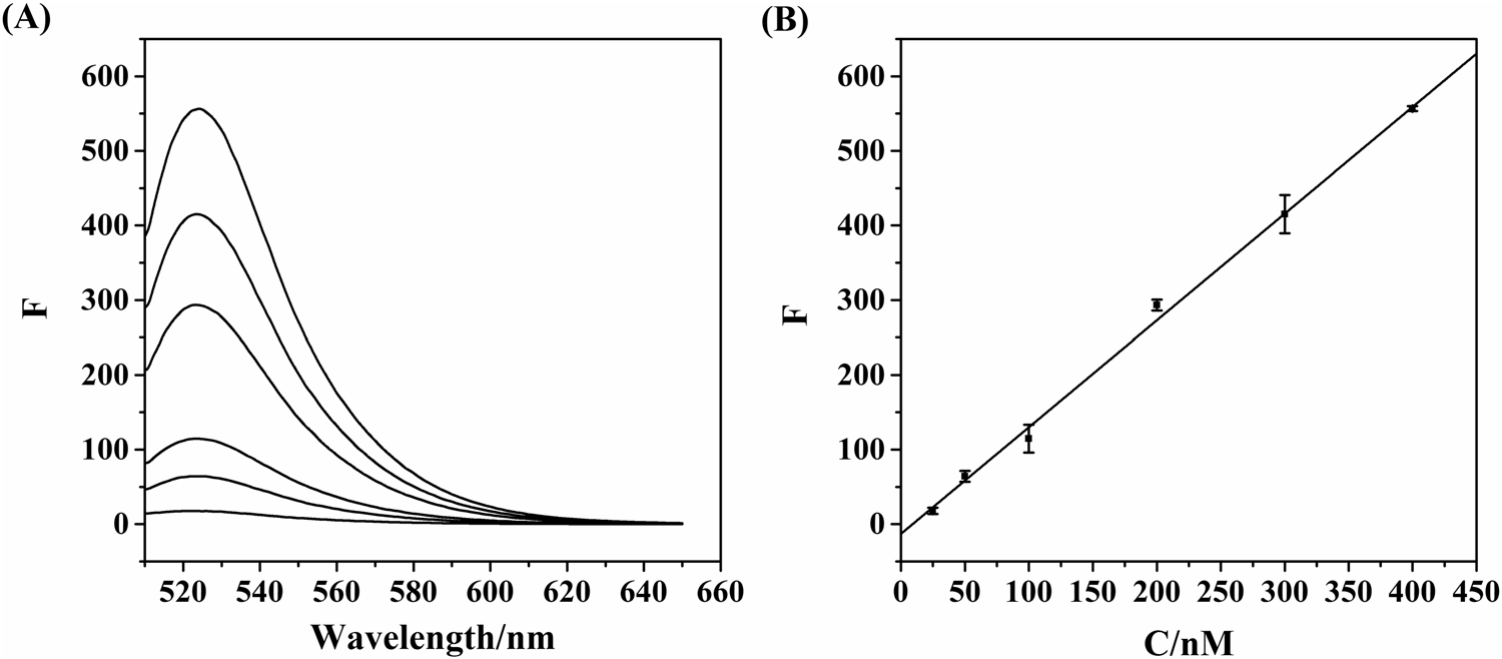
The proportional fluorescence restoration of the probe peptide FAM-PRG by the hybridization with target collagen peptide POG in PBS buffer. The fluorescence emission spectra were measured for FAM-PRG in the presence of various concentrations of POG (25, 50, 100, 200, 300, and 400 nM). (B) The fluorescence intensity monitored at 524 nm as a function of the concentration of POG.

The WS_2_/FAM-PRG platform was further examined to serve as a quantitative assay for collagen biomarkers. The probe peptide FAM-PRG was hybridized with various concentrations of the target collagen peptide POG. A linear relationship between the POG concentration and the fluorescence intensity of the platform was observed, while a larger concentration of POG led to stronger fluorescence (R^2^ = 0.99) (Fig. 5). This sensing platform displayed a broad linear range from 25 to 400 nM, with an accurate determination of the target collagen sequence POG as low as 8 nM. Collagen biomarkers were determined to be at ng/ml level in serum, and the antibody-based assay displayed a detection range of 1–500 ng/ml. This WS_2_/FAM-PRG platform has shown similar sensitivity for target peptides, while it requires a much simpler procedure than the ELISA assay.

### Quantitative detection of target peptide in biological fluids

**Figure 6:**
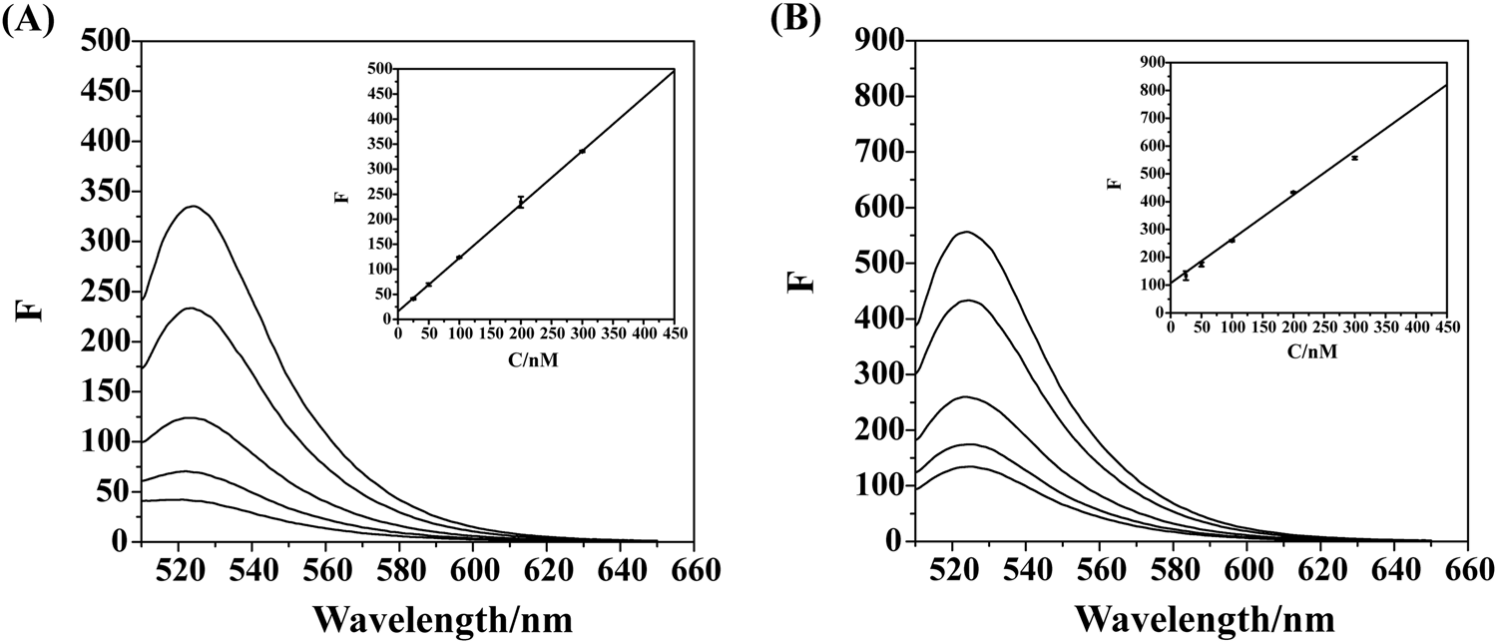
The linear fluorescence restoration of the probe peptide FAM-PRG by the hybridization with target collagen peptide POG in urine (**A**) and saliva (**B**) samples. The fluorescence emission spectra were measured for FAM-PRG in the presence of various concentrations of POG (25, 50, 100, 200, and 300 nM). Inset: The fluorescence intensity monitored at 524 nm as a function of the concentration of POG.

The assay to detect the target collagen peptide was further evaluated in human urine samples (Fig. 6A). As the concentration of peptide POG became larger, the fluorescence intensity of the sensing platform was proportionally increased (R^2^ = 0.99). The assay showed a broad linear range of 25–300 nM with a detection limit of 41 nM (Fig. 6A). A similar case was also observed for the assay in human saliva samples with a detection limit of 21 nM and a linear range of 25–300 nM (R^2^ = 0.99) (Fig. 6B). Though there might be other potentially interfering components in the biological fluids, a nice proportional relationship was well maintained between the concentration of peptide POG and the fluorescence intensity of the WS_2_/FAM-PRG platform. These results demonstrated that the newly developed WS_2_-based assay is capable to detect the target collagen sequence in complex media.

### Interferences

The interference by other proteins in this assay was examined (Fig. S8). The fluorescence intensity for the probe peptide FAM-PRG was measured in the WS_2_ platform in the presence of nonspecific proteins BSA, myoglobin, hemoglobin and protamine sulfate, respectively (striped bar). These proteins all resulted in much less florescence restoration than peptide POG, suggesting that the probe peptide was highly specific for the target collagen sequence. Furthermore, the fluorescence intensity of the hybridized mixture of probe peptide FAM-PRG and target peptide POG was measured in the WS_2_ platform in the absence and presence of nonspecific proteins (gray bar). The hybridized peptide mixtures with or without the nonspecific proteins displayed similar fluorescence recovery. These results proved that the WS_2_-based system could behave as a sensitive and selective platform for the detection of target collagen peptide with little interferences from other proteins.

## Conclusions

Transition metal dichalcogenides such as WS_2_ and MoS_2_ are newly discovered graphene analogs with superior properties. The selective adsorption of single stranded DNA by WS_2_ and MoS_2_ nanomaterials has been proved to be a valuable strategy to construct a variety of fluorescent biosensors^20, 21, 47^. However, little is understood between the interaction of other biomolecules such as peptides with WS_2_ and MoS_2_^21^. Herein we have explored peptides as probe biomolecules on WS_2_- and MoS_2_- based biosensing platforms.

We have first discovered that amino acid Arg can be distinctively adsorbed onto the surface of WS_2_ and MoS_2_ by investigating the interaction of amino acids with WS_2_ and MoS_2_ nanosheets. We have further revealed that the WS_2_ and MoS_2_ nanosheets show a selective adsorption of our specially constructed fluorescent Arg-rich probe peptide, leading to the fluorescence quenching of the dye; while the hybridization of the probe peptide and the target collagen sequence recovers the fluorescence. The established WS_2_-based platform is highly specific to the target collagen peptide with little interference from other proteins, and it can be conveniently employed in the quantitative detection for complex biological fluids.

These WS_2_- and MoS_2_- based peptide biosensing platforms have many advantages such as easy preparation and manipulation, and can be expanded to construct other novel peptide probes for the detection of various critical biomolecules. The coupling of transition metal dichalcogenides nanosheets with other biomolecules such as peptides opens new opportunities for the construction of multifunctional WS_2_- and MoS_2_- based biosensors with versatile features, which have great potential in broad biomedical applications.

## Electronic supplementary material

Supplementary Information
