# Supplementary material for: WS2 and MoS2 biosensing platforms using peptides as probe biomolecules
Source: Sci Rep. 2017 Aug 31;7:10290. doi: 10.1038/s41598-017-10221-4 (PMC5579024; doi:10.1038/s41598-017-10221-4)
Supplement: Supplementary file 1 — Supplementary Information [file 41598_2017_10221_MOESM1_ESM.doc]

Supporting information

WS_2_ and MoS_2_ biosensing platforms using peptides as probe biomolecules

*Xiuxia Sun, Jun Fan, Caihong Fu, Linyan Yao, Sha Zhao, Jie Wang, Jianxi Xiao*


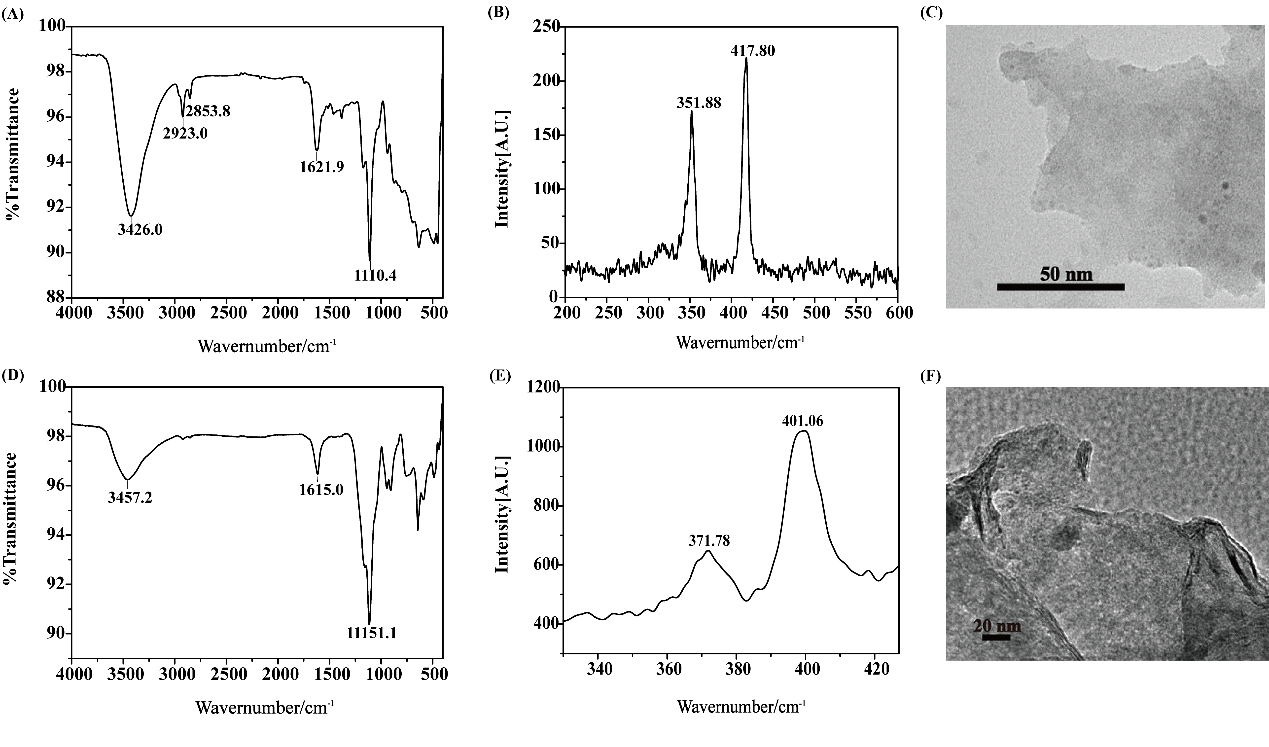

**Figure S1.** Characterization of WS_2_ and MoS_2_. FT-IR (A), Raman (B) and TEM (C) graphs of WS_2_. FT-IR (D), Raman (E) and TEM (F) graphs of MoS_2_.





**Figure S2.** The concentration ratio (C/C_o_) of the tested amino acids after and before the incubation with MoS_2_. C is the concentration of the tested amino acids after the incubation with MoS_2_, representing the un-absorbed amino acid by MoS_2_; C_o_ is the concentration of the tested amino acids before the incubation with MoS_2_, representing the total amino acid.


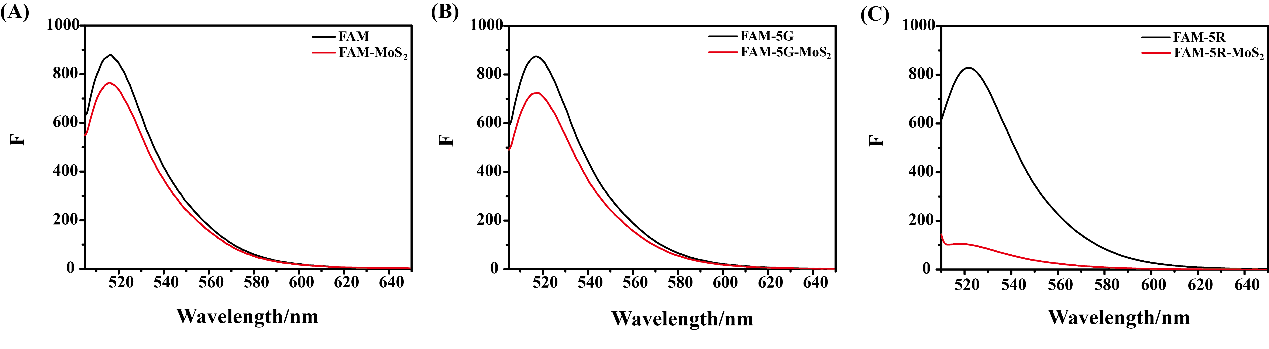


**Figure S3.** The fluorescence quenching of FAM, FAM-5G and FAM-5R by MoS_2_. The fluorescence intensities of FAM (A), FAM-5G (B) and FAM-5R (C) were measured in the presence (red) and absence (black) of MoS_2_ (A-C).


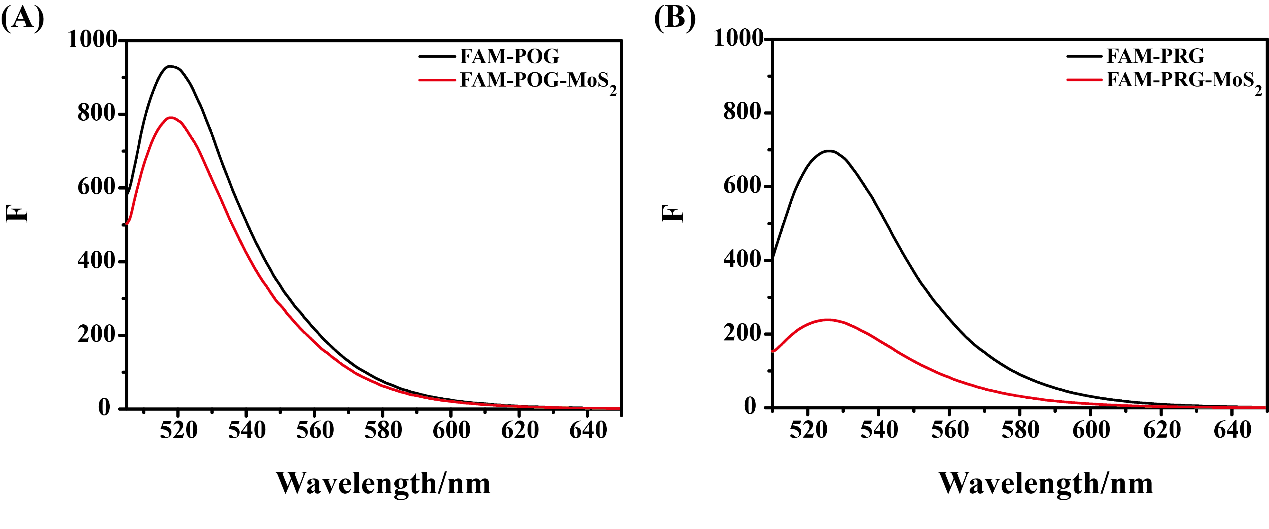


**Figure S4**. The fluorescence quenching of FAM-POG and FAM-PRG by MoS_2_. The fluorescence intensities of FAM-POG (A), and FAM-PRG (B) were measured in the presence (red) and absence (black) of MoS_2_ (A-B).


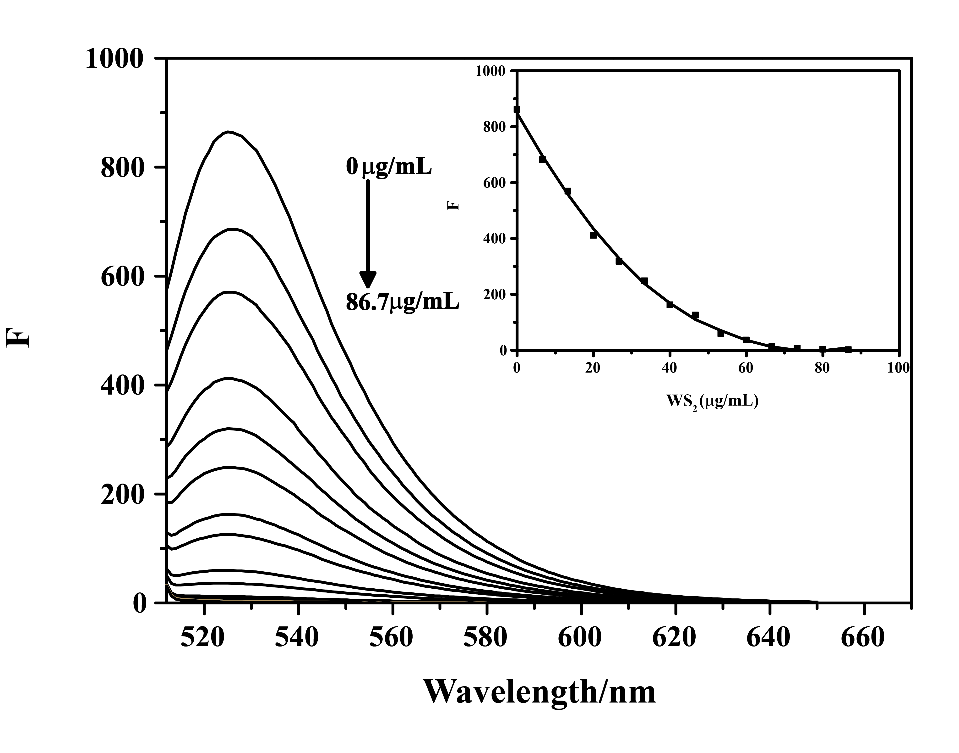


**Figure S5.** The fluorescence quenching of the probe peptide FAM-PRG by WS_2_. The fluorescence emission spectra were measured for FAM-PRG in the presence of various concentrations of WS_2_ (0, 6.7, 13.3, 20.0, 26.7, 33.3, 40.0, 46.7, 53.3, 60.0, 66.7, 73.3, 80, and 86.7 µg/mL). Inset: The fluorescence intensity monitored at 524 nm as a function of WS_2_ concentration.

**

**

**Figure S6.** The fluorescence restoration of the probe peptide FAM-PRG by the hybridization with the target collagen sequence POG using the WS_2_ (A) and MoS_2_ (B) platforms, respectively. The fluorescence emission spectra were measured for FAM-PRG without (black) or with (red) prior hybridization with peptide POG.





**Figure S7.** The effect of pH and salt on the fluorescence restoration of the probe peptide FAM-PRG by the target collagen sequence POG on the WS_2_ platform. The fluorescence intensities were measured for FAM-PRG hybridized (black) or unhybridized (red) with peptide POG at 524 nm under different pHs (A) and salt concentrations (B), respectively. Buffers with different pHs were prepared using 20 mM PBS, while different amounts of NaCl were added in 20 mM PBS buffer at pH 7.4.





**Figure S8**. The fluorescence restoration of the probe peptide FAM-PRG by nonspecific proteins. The fluorescence intensities were measured for the probe peptide FAM-PRG at 524 nm in the presence of nonspecific proteins BSA, myoglobin, hemoglobin and protamine sulfate, respectively (striped bar). The fluorescence restoration of the probe peptide FAM-PRG by the target peptide POG was determined in the absence and presence of nonspecific proteins (gray bar).
